# RNA-seq analysis reveals significant transcriptome changes in turbot (*Scophthalmus maximus*) suffering severe enteromyxosis

**DOI:** 10.1186/1471-2164-15-1149

**Published:** 2014-12-19

**Authors:** Diego Robledo, Paolo Ronza, Peter W Harrison, Ana Paula Losada, Roberto Bermúdez, Belén G Pardo, María José Redondo, Ariadna Sitjà-Bobadilla, María Isabel Quiroga, Paulino Martínez

**Affiliations:** Departamento de Genética, Facultad de Biología (CIBUS), Universidad de Santiago de Compostela, Santiago de Compostela, 15782 Spain; Departamento de Ciencias Clínicas Veterinarias, Facultad de Veterinaria, Universidad de Santiago de Compostela, Lugo, 27002 Spain; Department of Genetics, Evolution and Environment, University College London, London, UK; Departamento de Anatomía y Producción Animal, Facultad de Veterinaria, Universidad de Santiago de Compostela, Lugo, 27002 Spain; Departamento de Genética, Facultad de Veterinaria, Universidad de Santiago de Compostela, Lugo, 27002 Spain; Instituto de Acuicultura Torre de la Sal (IATS-CSIC), Ribera de Cabanes, Castellón, 12595 Spain

**Keywords:** RNA-seq, Transcriptome, Turbot, *Enteromyxum scophthalmi*, Enteromyxosis, Immune response, Apoptosis, Erythropoiesis, Cytoskeleton, Digestive function

## Abstract

**Background:**

Enteromyxosis caused by the intestinal myxozoan parasite *Enteromyxum scophthalmi* is a serious threat for turbot (*Scophthalmus maximus*, L.) aquaculture, causing severe catarrhal enteritis leading to a cachectic syndrome, with no therapeutic options available. There are still many aspects of host-parasite interaction and disease pathogenesis that are yet to be elucidated, and to date, no analysis of the transcriptomic changes induced by *E. scophthalmi* in turbot organs has been conducted. In this study, RNA-seq technology was applied to head kidney, spleen and pyloric caeca of severely infected turbot with the aim of furthering our understanding of the pathogenetic mechanisms and turbot immune response against enteromyxosis.

**Results:**

A huge amount of information was generated with more than 23,000 identified genes in the three organs, amongst which 4,762 were differently expressed (DE) between infected and control fish. Associate gene functions were studied based on gene ontology terms and available literature, and the most interesting DE genes were classified into five categories: 1) immune and defence response; 2) apoptosis and cell proliferation; 3) iron metabolism and erythropoiesis; 4) cytoskeleton and extracellular matrix and 5) metabolism and digestive function. The analysis of down-regulated genes of the first category revealed evidences of a connexion failure between innate and adaptive immune response, especially represented by a high number of DE interferon-related genes in the three organs. Furthermore, we found an intense activation of local immune response at intestinal level that appeared exacerbated, whereas in kidney and spleen genes involved in adaptive immune response were mainly down-regulated. The apoptotic machinery was only clearly activated in pyloric caeca, while kidney and spleen showed a marked depression of genes related to erythropoiesis, probably related to disorders in iron homeostasis. The genetic signature of the causes and consequences of cachexia was also demonstrated by the down-regulation of the genes encoding structural proteins and those involved in the digestive metabolism.

**Conclusions:**

This transcriptomic study has enabled us to gain a better understanding of the pathogenesis of enteromyxosis and identify a large number of DE target genes that bring us closer to the development of strategies designed to effectively combat this pathogen.

**Electronic supplementary material:**

The online version of this article (doi:10.1186/1471-2164-15-1149) contains supplementary material, which is available to authorized users.

## Background

Turbot (*Scophthalmus maximus*, L.) is a marine flatfish which has been intensively cultured in Europe for more than 25 years. Turbot aquaculture production currently accounts for over 10,000 tons/year in Europe, and has rapidly increased in China during the last decade, reaching more than 60,000 tons in 2011 [1]. Although its entire life cycle is routinely carried out at farm facilities, major challenges related to growth rate and disease outbreaks are the main concerns for the turbot industry [2–5].

The myxozoan genus *Enteromyxum* includes three intestinal species, which all cause serious problems for seawater aquaculture. *Enteromyxum*’s virulence varies depending on the specific parasite-host interaction, ranging from highly pathogenic in some species pairs, such as *E. scophthalmi* infection of turbot, to chronic (*E. leei* in gilthead sea bream, *Sparus aurata*) or subclinical (*E. fugu* in tiger puffer, *Takifugu rubripes*) disease signs in others. *E. scophthalmi* is a serious threat for cultured turbot, that spreads rapidly in farm facilities due to direct fish-to-fish transmission, and causes a cachectic syndrome which eventually leads to death [6]. Diseased fish present anorexia, anaemia, weight loss, poor conversion rates and delayed growth, with mortality rates reaching up to 100% in many cases [6, 7]. The main histological changes are catarrhal enteritis and lymphohaematopoietic depletion of spleen and kidney, the severity of which increases with the progression of the infection [8]. Although some drugs have been able to lower the mortality rates [9], currently there is no effective treatment for enteromyxosis. Understanding the disease pathogenesis through the study of host-parasite interaction and turbot immune response is critical in order to develop effective treatments and apply preventive measures. Numerous recent studies have been focused on elucidating these processes, mainly through histopathological, immunoenzymatic and serological assays [10–16]. Nevertheless, while PCR-array and microarray based molecular profiling of gilthead sea bream response to *E. leei* has been recently published [17, 18], gene expression characterization of *E. scophthalmi*-infected turbot is lacking. Transcriptome analysis is an invaluable tool for the elucidation of the biological processes behind host-parasite interactions, and in the last decade this approach, mainly based upon microarrays, has been extensively used in fish immunology and pathology [19], and specifically in turbot for analyzing furunculosis and scuticociliatosis [20, 21]. Additionally, the identification of relevant immune gene variants conferring tolerance to parasites is essential in order to develop marker assisted selection programmes that can lead to increased resistance [17, 22]. RNA-seq is a powerful technique for the analysis of gene expression due to its higher sensitivity and specificity in comparison to microarrays, along with its ability to detect new genes, rare transcripts, alternative splice isoforms, and novel SNPs which can be used for association studies [23–25]. For these reasons it is rapidly becoming the technology of choice for transcriptomic studies [19, 26].

In this study, a gene expression analysis of *E. scophthalmi*-infected turbot was carried out using RNA-seq on the two major lymphohaematopoietic organs, head kidney and spleen, and also on the pyloric caeca, the target intestinal region where the parasite infection starts in this species [27]. Fish categorised with a severe infection were used with the aim of capturing the gene expression signatures associated with advanced stages of the disease as a first reference to investigate the genetic mechanisms underlying the pathogenesis of enteromyxosis. Our findings constitute the basis of future studies aimed at investigating resistance-related genes and associated genetic variants that could be applied in breeding programmes. This is the first study to tackle the molecular basis of lesion development and the immune response underlying enteromyxosis in turbot.

## Results

### Histopathology

Experimentally-infected (recipient, RCPT) fish selected for RNA-seq analysis presented catarrhal enteritis characterized by severe parasitic load along the entire gastrointestinal tract associated with moderate to severe inflammatory infiltrates and lining epithelium detachment. In most gut segments, apoptotic figures in both epithelium and lamina propria were observed, while signs of epithelium regeneration were sporadically annotated. Spleen and head kidney showed moderate to severe cellular depletion, with occasional observation of indicators of apoptosis. There were no significant histological changes in the other organs of recipient fish (RCPT) neither in any samples from control fish (CTRL).

### RNA-seq

**Figure 1:**
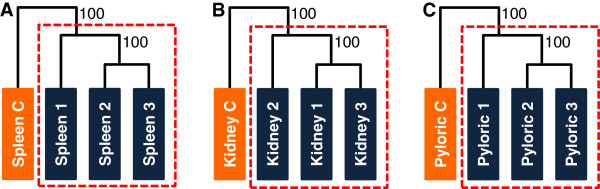
**Samples hierarchical clustering by organ.** Hierarchical clustering of all diseased and control samples for **A** Spleen, **B** Head kidney and **C** Pyloric caeca. Approximately unbiased *P*-values, computed by multi-scale bootstrap resampling, are displayed on branch nodes and clusters of samples with an approximately unbiased *P*-value > 0.95 are indicated with a dashed red box, indicating strong support.

A total of ~170 million 100 bp pair-end reads were sequenced, accounting for on average 15 million reads per sample post-filtering. Filtered reads were mapped to the turbot genome identifying a total of 54,864 transcripts and 23,063 genes in the three organs. The average number of raw, filtered and mapped reads for the samples of each organ are shown in Table 1. For each organ the control and infected samples were hierarchically clustered according to their transcript expression (Figure 1) confirming the correct classification of infected and control samples.

**Table 1:** **RNA-Seq sample statistics**

Sample	Raw reads	Trimmed reads	Aligned reads
Head kidney	17,431,026	15,929,258	14,321,389
Spleen	16,393,159	14,705,171	13,124,477
Pyloric caeca	17,094,324	15,341,340	9,963,567

Average raw reads obtained by Illumina sequencing, average trimmed reads remaining after filtering and average reads aligned to the turbot genome per sample are shown for each organ.

### Differential expression analysis

**Figure 2:**
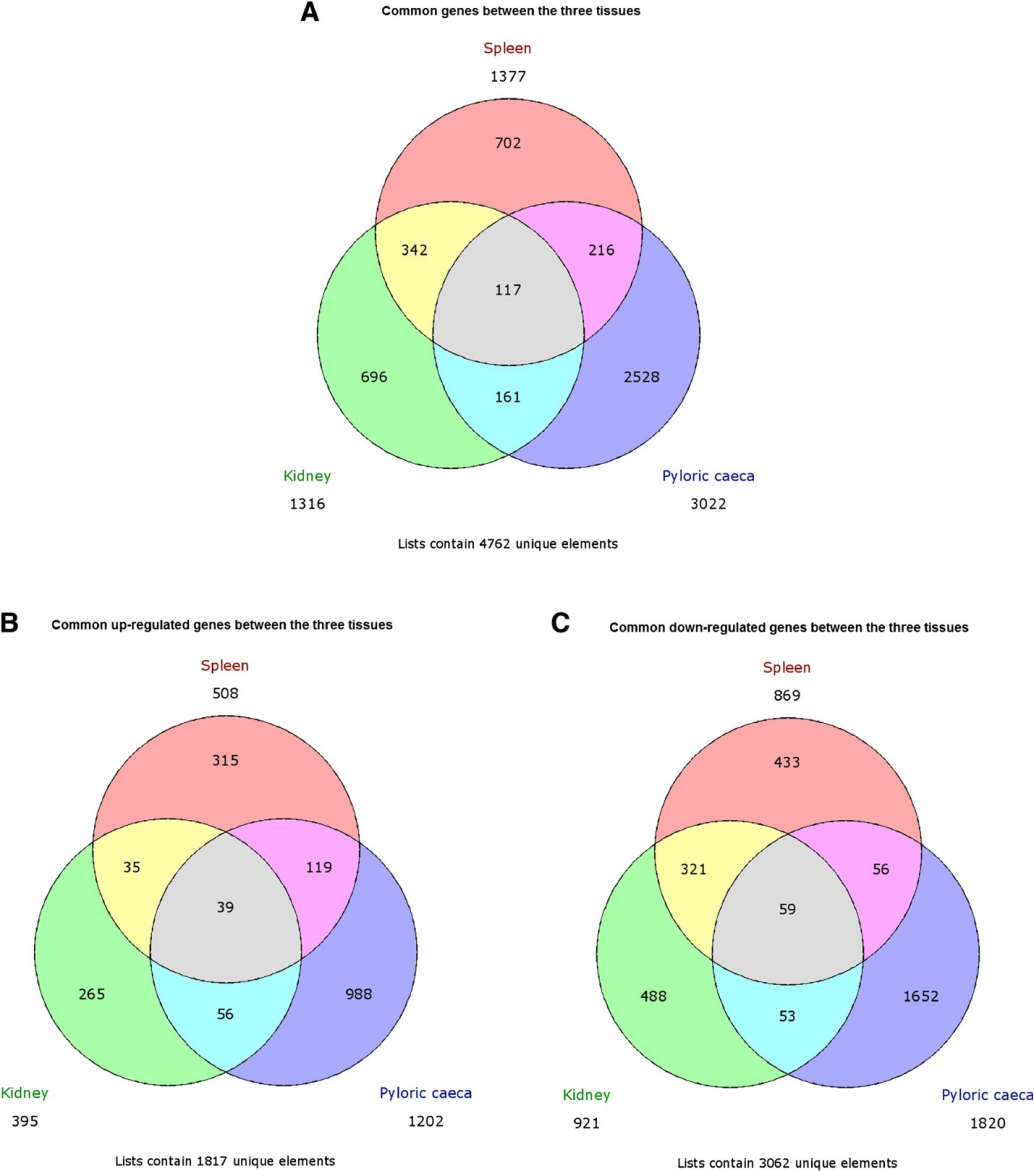
**DE genes Venn diagrams.** Venn diagrams of **A)** all DE genes, **B)** up-regulated DE genes, **C)** down-regulated DE genes in the three organs (head kidney, spleen and pyloric caeca) are shown. The total number of DE expressed genes in each tissue and the number of unique and common genes between them is displayed.

A total of 4,762 differentially expressed (DE) genes were identified across the three organs when comparing RCPT and CTRL fish. The number of DE genes (up- and down-regulated) for each organ and those shared between organs is shown in Figure 2. A high number of down-regulated genes (3,062) were detected, 68.5% more than up-regulated (1,817). Pyloric caeca showed the highest amount of DE genes, almost double that of the other two organs. The percentage of DE annotated transcripts was similar in the three organs: 44.1% in head kidney, 181 up-regulated and 400 down-regulated; 42.3% in spleen, 229 up-regulated and 353 down-regulated; and 46.9% in pyloric caeca, 562 up-regulated and 851 down-regulated. Log_2_ fold change (FC) values ranged from 11.26 to -11.18 in head kidney, from 13.29 to -12.83 in spleen, and from 12.13 to -15.18 in pyloric caeca.

#### Common DE genes between the three organs

A group of 117 DE genes were shared between the three organs. Among them, 48 were successfully annotated: 11 up- and 26 down-regulated, and 11 either up- or down-regulated depending on the organ (Additional file 1: Table S1, ST1).

Amongst the shared up-regulated genes, some were involved in innate immune response and antigen presentation such as *IL4I1*, involved in the lysosomal processing and presentation of antigens; *ALOXE3*, an enzyme that participates in leukotrienes metabolism; and *MASP3b*, which plays a prominent role in the activation of the lectin complement pathway.

Amongst the down-regulated genes, two sets were of particular interest, one directly involved in immune response and the other related to cell and tissue structure disruption. The first group included several interferon (IFN)-related genes involved in antiviral immune response, such as *IRF7*, *Gig2, IFIT-1, cHERC5* and *IFI44*. Other two interesting genes were related to major histocompatibility complex class I molecules (MHC-I), involved in the presentation of intracellular-derived antigens, and *CAT*, which encodes an essential antioxidant enzyme for cell protection against oxidative damage. The second set included several genes related to cytoskeleton: *FMNL1* (cytoskeletal organization and cell morphology and motility); *TMOD4* (geometry of the membrane skeleton); *GRXCR1* (architecture of actin filament-rich structures); and *SNPH* (microtubule-associated protein). *COL1A2*, involved in extracellular matrix structure and organization, was also found in this group.

A group of 12 up-regulated genes in pyloric caeca, but down-regulated in head kidney and spleen, included three genes which promote apoptosis (*KCNN3*, *EGR* and *TNFRSF10b*) and the immunoglobulin light chain, which plays a key role in the adaptive immune response.

The only up-regulated gene in pyloric caeca and head kidney, but down-regulated in spleen was the complement component C3, essential for the activation of the complement pathway. Finally, the only gene up-regulated in spleen and head kidney and down-regulated in pyloric caeca was aminopeptidase n, which encodes an enzyme that participates in the final digestion of peptides, but also in processing other peptide molecules such as hormones, neuropeptides and MHC class II-bound antigen peptides.

#### DE genes classification and organ-specific expression

**Figure 3:**
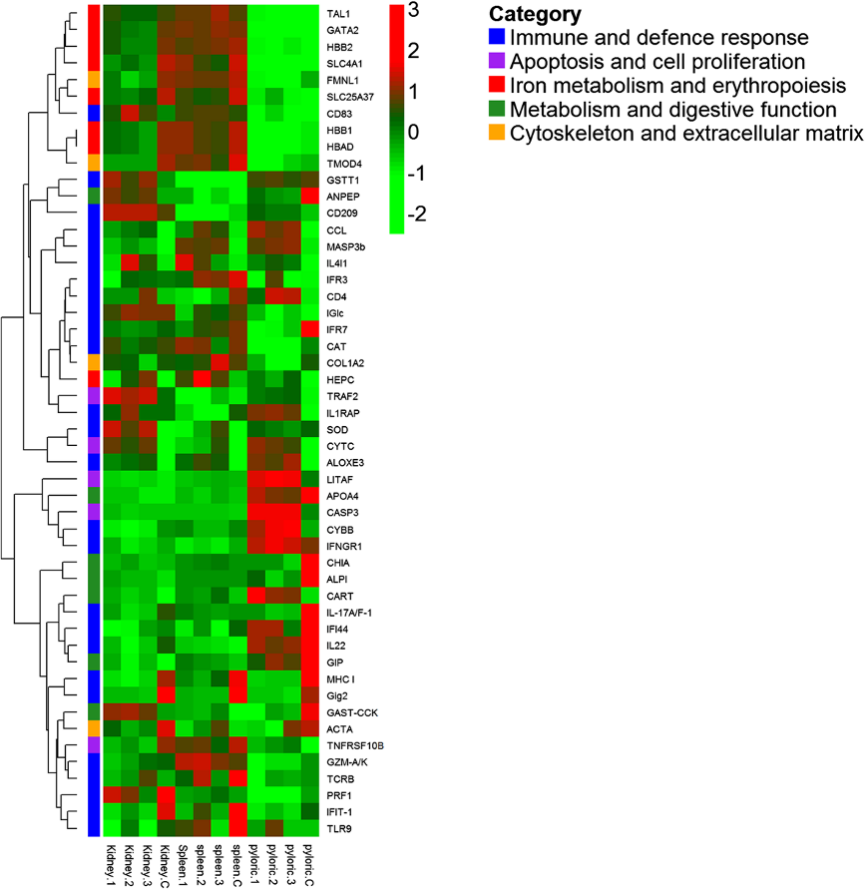
**Heatmap of 50 selected genes.** Heatmap of the fifty selected genes presented in Table 2. Displayed are EdgeR [101] normalized counts for each sample and gene. Sample names are displayed at the bottom of the figure whilst gene symbols are shown to the right and have been hierarchically clustered according to their pearson correlation. The category assigned to each gene is also shown with a color code.

We inspected the list of organ-specific DE transcripts and made a selection of the most interesting genes, which were grouped in five key broad functional categories based on GO term characterisation and on the current literature in the field. The chosen categories were 1) immune and defence response, 2) apoptosis and cell proliferation, 3) iron metabolism and erythropoiseis, 4) metabolism and digestive function and 5) cytoskeleton and extracellular matrix. The selected genes and the group to which they belong to are listed in Additional file 2: Table S2 (ST2) for head kidney, Additional file 3: Table S3 (ST3) for spleen and Additional file 4: Table S4 (ST4) for pyloric caeca. A selection of the 50 most relevant DE genes from these five categories is presented in Table 2 and their expression shown in a heatmap (Figure 3).

**Table 2:** **Selection of the 50 most relevant DE genes**

Gene	Short name	Log-FC head kidney	Log-FC spleen	Log-FC pyloric caeca	Category	Associated function
MHC class I antigen	MHC I	-6.3	-4.9	-5.4	1	Antigen processing and presentation
Interferon regulatory factor 7	IFR7	-1.8	-1.6	-3.6	1	Positive regulation of interferons production
Interferon-induced protein 44	IFI44	-3.2	-3.6	-2.2	1	Interferon-inducible protein
Interferon-induced protein with tetratricopeptide repeats 1	IFIT-1	-4.7	-5.0	-2.1	1	Interferon-inducible protein
Gig2-like protein	Gig2	-10.4	-12.8	-7.4	1	Interferon-inducible protein
Interferon gamma receptor alpha chain	IFNGR1	-2.7	-4.4	--	1	Regulation of interferon-gamma-mediated signalling pathway
Interferon regulatory factor 3	IFR3	-1.8	-2.1	--	1	Positive regulation of interferons production
Toll-like receptor 9	TLR9	-3.0	-3.0	--	1	Innate immune response; Positive regulation of interferons production
Mannose-binding lectin-associated serine protease-3b	MASP3b	7.6	4.9	4.5	1	Complement activation
Epidermis-type lipoxygenase 3-like	ALOXE3	5,7	6.8	10.3	1	Leukotriene metabolic process
L-amino-acid oxidase-like	IL4I1	4.8	5.9	9.5	1	Innate immune response
Interleukin-17a f-1	IL-17A/F-1	--	--	-3.8	1	Inflammatory response
Interleukin-22	IL22	-4.5	--	-1.7	1	Inflammatory response
CD83 antigen	CD83	--	-1.8	2.5	1	Defence response
CD209 antigen-like	CD209	--	-4.3	2.2	1	Innate immune response
Cytochrome b-245 heavy chain	CYBB	-4.0	--	3.7	1	Inflammatory response
CC chemokine	CCL	--	1.9	4.0	1	Inflammatory response
Interleukin-1 receptor accessory protein	IL1RAP	--	-4.2	2.6	1	Inflammatory response
Lipopolysaccharide-induced tumor necrosis factor-alpha factor	LITAF	--	-1.6	6.2	1,2	Regulation of cytokine production-Apoptotic process
Immunoglobulin light chain	IGlc	-1.8	-2.0	2.7	1	Antigen binding
T-cell surface glycoprotein cd4	CD4	--	-1.6	--	1	T cell receptor signalling pathway
T-cell receptor beta chain	TCRB	--	-2.0	--	1	T cell receptor signalling pathway
Perforin-1-like	PRF1	-3.2	--	--	1	T cell-mediated cytolysis
Granzyme A/K	GZM-A/K	-1.7	--	--	1	T cell-mediated cytolysis
Catalase	CAT	-2.0	-1.8	-2.5	1	Hydrogen peroxide catabolic process
Superoxide dismutase	SOD	--	--	-2.1	1	Removal of superoxide radicals
Glutathione s-transferase theta-1	GSTT1	--	--	-2.0	1	Oxidation-reduction process
Caspase-3-like	CASP3	--	--	5.5	2	Positive regulation of apoptotic process
Cytochrome c	CYTC	2.5	--	4.3	2	Apoptotic DNA fragmentation
TNF receptor-associated factor 2-like	TRAF2	--	--	3.6	2	Regulation of apoptotic process
Tumor necrosis factor receptor superfamily member 10b-like	TNFRSF10B	-3.2	-2.2	2.6	2	Regulation of apoptotic process
Hemoglobin subunit alpha-d	HBAD	-5.2	-3.5	--	3	Oxygen transport
Hemoglobin subunit beta-2	HBB2	-4.7	-3.8	--	3	Oxygen transport
Hemoglobin subunit beta-1	HBB1	-4.6	-3.2	--	3	Oxygen transport
Band 3 anion exchange protein	SLC4A1	-6.6	-4.5	--	3	Erythrocytes differentiation
Gata-binding factor 2-like	GATA2	-3.5	-2.2	--	3	Erythrocytes differentiation
Mitoferrin-1	SLC25A37	-3.6	-3.5	--	3	Erythrocytes maturation
T-cell acute lymphocytic leukemia protein 1	TAL1	-2,8	-1,6	--	3	Erythrocytes differentiation- Erythrocytes maturation
Hepcidin	HEPC	2.7	2.0	--	3	Iron metabolism
Aminopeptidase n	ANPEP	2.2	7.3	-9.2	4	Protein metabolic process
Intestinal-type alkaline phosphatase 1	ALPI	--	--	-11.7	1,4	Metabolic process
Acidic mammalian chitinase	CHIA	--	--	-14.1	4	Carbohydrate metabolic process
Apolipoprotein a-iv precursor	APOA4	--	--	-6.5	4	Lipoprotein metabolic process
Gastric inhibitory polypeptide	GIP	--	--	-3.1	4	Response to nutrient levels
Cocaine- and amphetamine-regulated transcript	CART	--	--	6.7	4	Negative regulation of appetite
Gastrin cholecystokinin-like peptide	GAST-CCK	--	--	-4.4	4	Digestion
Collagen alpha-2 chain	COL1A2	-2.0	-1.5	-2.4	5	Extracellular matrix structural constituent
Tropomodulin 4	TMOD4	-5.6	-3.9	-2.3	5	Tropomyosin binding
Formin-like protein 1-like	FMNL1	-4.0	-3.0	-2.8	5	Actin cytoskeleton organization
Alpha actin	ACTA	-3.9	--	-2.7	5	Skeletal muscle fiber development

Statistically significant fold changes are shown for each organ for 50 relevant genes associated with enteromyxosis. Categories: 1) Immune and defence response; 2) Apoptosis and cell proliferation; 3) Iron metabolism and erythropoiesis; 4) Metabolism and digestive function; 5) Cytoskeleton and extracellular matrix. Non significant differences have been marked as “--”.

#### Immune and defence response

A large number of genes related to this function were found in the three organs, but, while the number of down-regulated genes was comparable, pyloric caeca showed the most numerous group of up-regulated genes. This included chemokines, chemokine receptors, immunoglobulin chains, interleukins and several other genes involved in both innate and adaptive immune response. In particular, a broad representation of components of the inflammatory reaction pathway like *G-CSFR*, immune-responsive gene-1, p- and e-selectin, as well as the transcription factors *AP-1* and *CEBPB*, was detected. Moreover, genes such as *LITAF*, which promotes the expression of the pro-inflammatory cytokine *TNF-α*, and *CYBB*, a superoxide-generating enzyme of phagocytes, were up-regulated in pyloric caeca but down-regulated in spleen and head kidney, respectively. Lymphoid organs, spleen and head kidney, showed a similar number of up- and down-regulated genes involved in inflammation and acute-phase response, including shared up-regulated genes like hepcidin, heat shock proteins and prostaglandin synthases. On the other hand, several genes related to B and T cells (such as those encoding immunoglobulin molecules and the T cell-related proteins *CD4*, *TCRB*, granzyme and perforin) were down-regulated in these organs. Moreover, in spleen there was a depression of two genes considered to be markers for dendritic cells, *CD83* and *CD209*, which were, conversely, up-regulated in pyloric caeca. Spleen and pyloric caeca, in turn, showed a common up-regulation of the C-type lectin *MRC1,* while other two members of this family were up-regulated, but only in spleen (*MASP1*) or in pyloric caeca (*CLEC4M*).

Noticeably, more genes related to IFNs were identified among down-regulated genes, like *Gig1*, *IFNGR1* and *IFR3* in head kidney and spleen or *GVINP1* and *IRF4* in spleen and pyloric caeca. Moreover, the *TLR9*, also involved in defence against intracellular pathogens, was down-regulated in spleen and head kidney.

Also, some regulated genes involved in Th17 cells response were detected, particularly the down-regulation of Th17 cytokines: *IL22* in head kidney and pyloric caeca and *IL17* and its receptor in pyloric caeca. In this organ, it was also remarkable the down-regulation of four genes related to anti-oxidant defence (*MRSA*, *SOD*, *GSTT1* and *TXNDC17*) and the up-regulation of several genes involved in tissue remodelling and repair (e.g. *MMP13*, *PLAT*, *FGF10*, *VNT*). Spleen and head kidney, as well, showed up-regulation of *HGF*, a cytokine acting in tissue repair but also in modulation of immune response, and of two *HGF*-related genes (*HGFR* in spleen and *HGFAC* in head kidney).

Finally, a few genes known to be induced under hypoxic and/or oxidative stress conditions were found to be up-regulated, including the angiopoietin related proteins showing an increase between 4 and 7.4 FC in the three organs, and *HIGD1A*, the adrenomedullin genes and a cytochrome c oxidase mitochondrial subunit in pyloric caeca and head kidney.

#### Apoptosis and cell proliferation

Apoptosis and cell proliferation DE genes were found mainly in pyloric caeca. Several genes participating in the apoptotic process, especially the caspase-3 (FC = 5.5) and cytochrome c (FC = 4.3), which are essential players in the execution phase of apoptosis, were up-regulated. In general, in this organ we found more pro- than anti-apoptotic genes, but also other genes involved in cell proliferation, such as the *PCNA*, *FGF10* and cyclins b1 and a2, were up-regulated. In head kidney and spleen a few genes belonging to this group, like the pro-apoptotic cytochrome c in head kidney and clusterin in spleen were up-regulated.

#### Iron metabolism and erythropoiesis

In head kidney and spleen, the main lymphohaematopoietic organs in teleosts, we observed down-regulation of several genes related to haematopoiesis. The expression of genes involved in erythrocyte maturation and differentiation, like *TAL1*, *GATA2* and mitoferrin-1, was depressed in both organs, and we also observed a dramatic decrease in the expression of genes related to oxygen transport. For example, haemoglobin subunit alpha-d and haemoglobin subunit beta-2 showed a -5.2 and a -4.7 FC, respectively, in head kidney, and a -3.5 and -3.8 in spleen. The band 3 anion transport protein gene, the major glycoprotein of the erythrocyte membrane, also suffered a notable down-regulation in head kidney (FC = -6.6) and spleen (FC = -4.6). On the other hand, two genes related to iron homeostasis, hepcidin (up-) and ferritin heavy subunit (down-), were regulated in both organs.

#### Metabolism and digestive function

This group of genes was analyzed in pyloric caeca to evaluate intestinal function during enteromyxosis, and were mainly down-regulated. Most of these genes showed high expression in the control sample, while its expression was practically undetectable in infected individuals. That was the case of the digestive enzymes *CHIA* (-14.1), *ALPI* (-11.7), *CYP7A1* (-9.1) or *CPO* (-6.0). Also the *FABP2* and *APOA4* genes, involved in lipid metabolism, showed very highly expression in control samples, but -4.1 and -6.5 FCs, respectively, in infected samples. Moreover, there was a depression of genes induced by food intake (*GIP*, *CCK2*, gastrin-cholecystokinin-like peptide) and of the gene coding for the galanin type I receptor, an orexigenic petide. On the other hand, two anorexigenic genes (*CART* and *CGRP*) were up-regulated.

#### Cytoskeleton and extracellular matrix

Several myosin, collagen, actin, tubulin, coronin and spectrin genes, were down-regulated in the three organs. Pyloric caeca and head kidney exhibited the highest number of down-regulated genes. Of particular interest were collagen alpha-1, alpha actin and the different myosin genes, that were abundantly expressed in control samples, showing FCs ranging from -6.1 (myosin heavy chain) to -1.8 (collagen alpha-1). Likewise, the *TPM4*, which was highly expressed in spleen and pyloric caeca of CTRL fish, was down-regulated in RCPT samples.

## GO enrichment analysis

**Figure 4:**
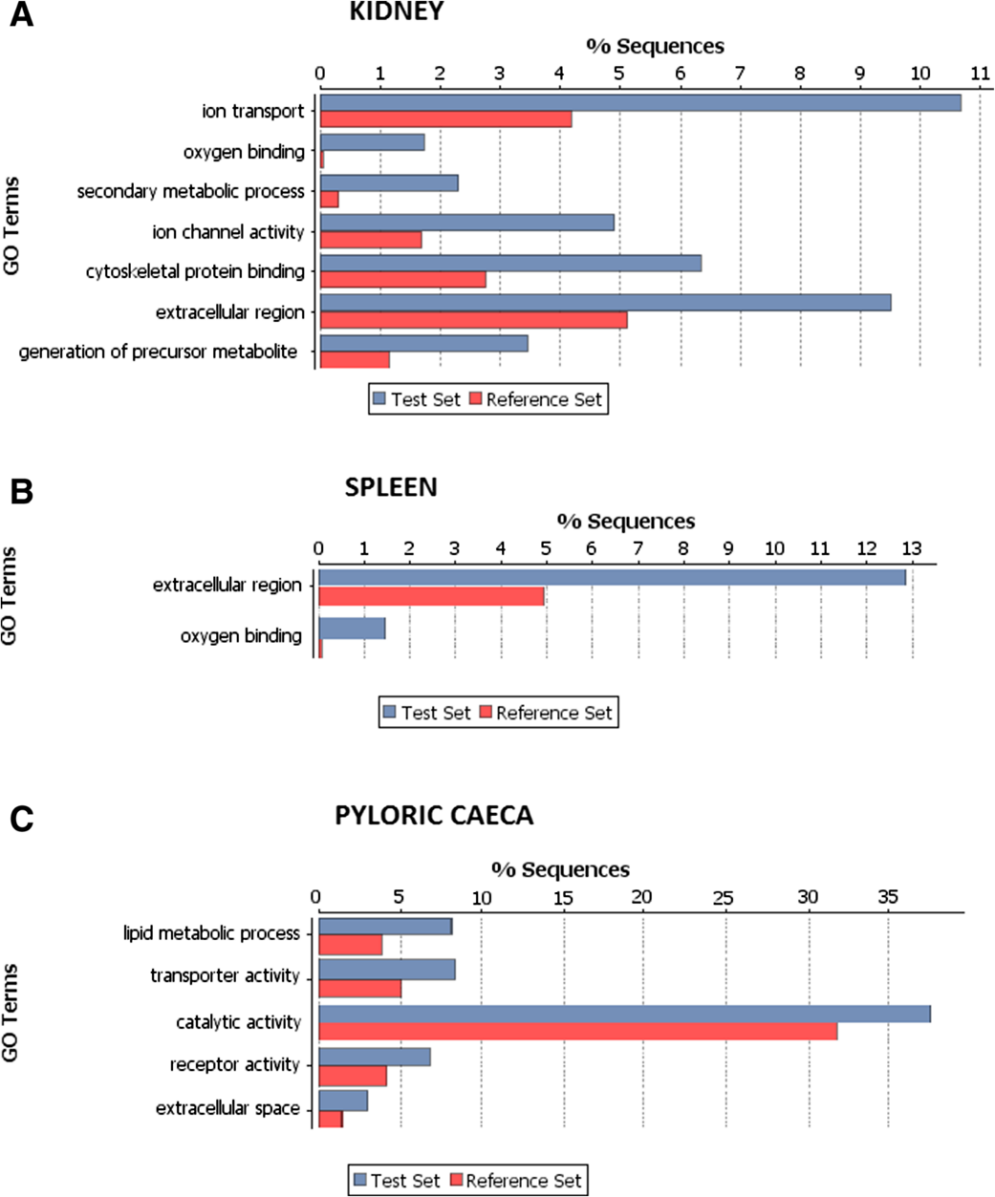
**GO terms enrichment.** GO enrichment (p < 0.01 FDR corrected) for DE genes in **A)** head kidney, **B)** spleen and **C)** pyloric caeca. The percentage of sequences with the associated GO term present in the full organ transcriptome is shown in blue, while the percentage of sequences with the GO term in the DE gene group is shown in red.

The full transcriptome of the three organs was annotated and GO terms for each sequence were obtained. A Fisher exact test (FDR corrected p-value = 0.05) was used to compare DE sequences with the background transcriptome to obtain the enriched GO terms for each organ (Figure 4). Oxygen binding was clearly overrepresented in both spleen and head kidney of RCPT fish, likely indicating alterations in the erythrocyte machinery, as mentioned earlier. Lipid metabolism and catalytic activity were enriched categories in pyloric caeca, which might evidence problems in the digestive function. Extracellular space or extracellular region GO terms were present in all three organs. GO enrichment was also performed for up-regulated and down-regulated genes separately, obtaining an additional up-regulated GO category in spleen, peptidase activity, and in pyloric caeca, cell cycle.

## Discussion

**Figure 5:**
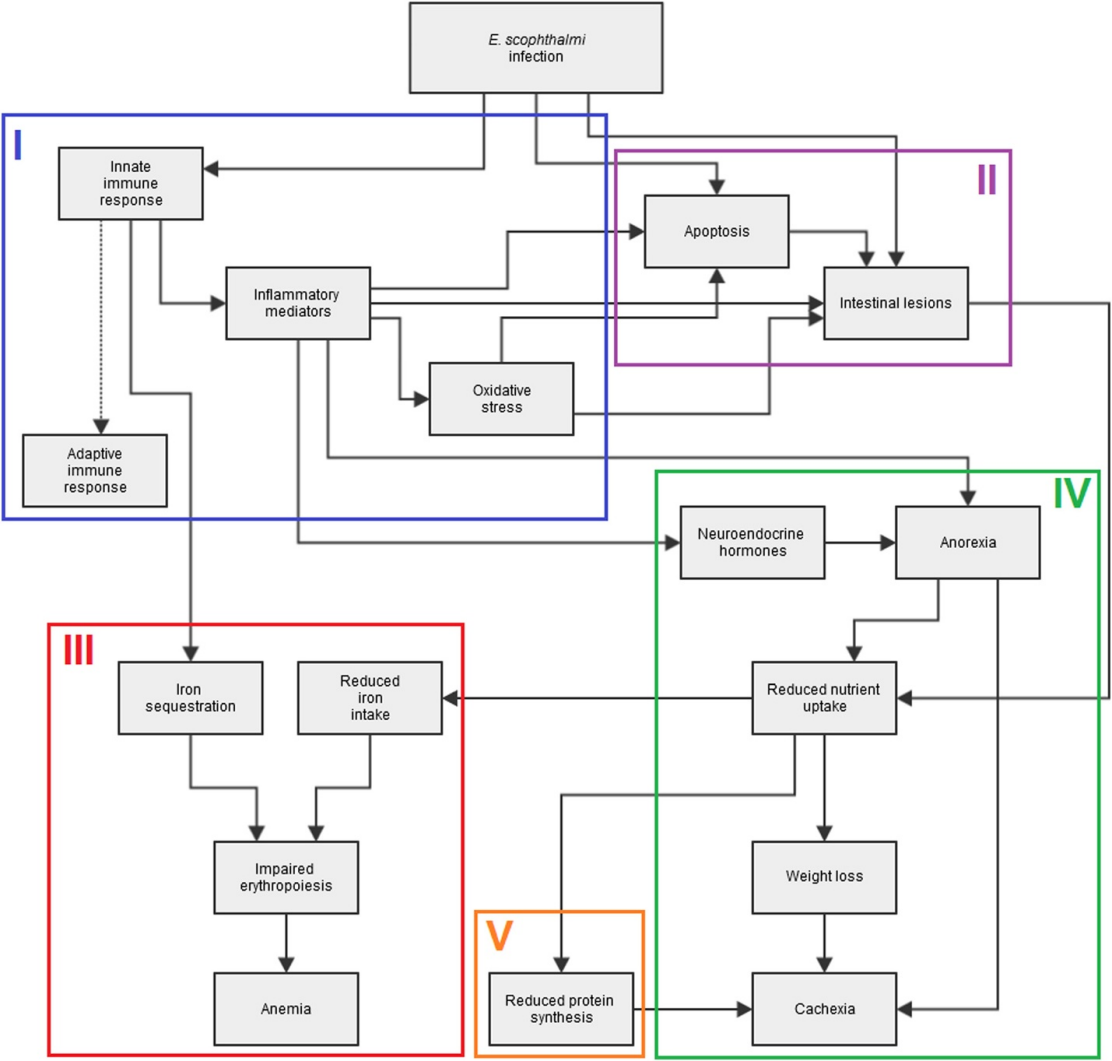
**Enteromyxosis flowchart.** Flowchart showing the main processes involved in severe turbot enteromyxosis. The flowchart has been divided in five sections according to the most representative processes occurring during Enteromyxum infection: I, blue, immune and defence response; II, purple, apoptosis and cell proliferation; III, red, iron metabolism and erythropoiesis; IV, green, metabolism and digestive function; V, orange, cytoskeleton and extracellular matrix.

This is, to our knowledge, the first report of a RNA-seq transcriptomic analysis applied to the study of a fish-parasite model. We investigated turbot at an advanced enteromyxosis stage, selected on the basis of histopathological evaluation. This approach allows the analysis of fish with a more uniform health status, minimizing interindividual variation, and consequently, enabling a more consistent identification of regulated genes on a reduced number of animals. This study advances our understanding of how the *E. scophthalmi* infection develops and the determination of the clinical signs and lesions characteristic of infection in turbot. Figure 5 depicts the cascade of events leading to severe enteromyxosis in turbot considering in particular the transcriptomic changes found in the current study.

We found far more DE genes in pyloric caeca (3022) than in either kidney or spleen (1316 and 1377, respectively). This is perhaps not that surprising since the intestine is the target tissue of *Enteromyxum spp.* infection and the lesions caused by the disease are mostly restricted to the gastrointestinal tract. Additionally, the most characteristic clinical signs of the disease, such as weight loss and anorexia, are attributable to the alteration of the normal physiology of the digestive system. Furthermore, spleen and kidney shared 321 down-regulated genes, an interesting result which can be attributed to the common lymphohaematopoietic functions and the cellular depletion observed in both organs in late stages of the disease [8].

### Immune and defence response

The defence response of turbot against *E. scophthalmi* was characterized by the activation of the innate immune response, but it seems that different elements acting in bridging innate and adaptive response are failing, and an inadequate onset of the adaptive immune response was noticed. The transcriptomic changes detected were especially intense in pyloric caeca, corresponding, and likely contributing, to the severe histological changes observed at tissue level.

Numerous regulated genes belonging to immune and defence response were found in the three organs analyzed. The number of up-regulated genes in pyloric caeca far exceeded the down-regulated ones, while this is not observed in kidney and spleen. Among the three DE genes related to the innate immune response shared by the three organs (*ALOXE3*, *IL4I1* and *MASP3b*), the overexpression of *MASP3b*, which mediates the activation of the complement lectin pathway, is of particular interest [28–30]. This is also the function of MASP1, overexpressed in spleen. The complement lectin pathway is considered the most ancient route of complement activation [31, 32], with a well-demonstrated role in several parasitic diseases [33–36]. Interestingly, the third component of the complement system, C3, which plays a central role supporting the activation of the three complement pathways [37] was up-regulated in head kidney and pyloric caeca, but down-regulated in spleen. This could be related to the changes also observed in components of the classical pathway (overexpression of C1q receptor in spleen and pyloric caeca, but depression of C1q-like protein 4 and protein 2 in pyloric caeca), probably as a rebound effect after a previous increase, or due to a progressive exhaustion of the complement system, hypothesized in late stages of enteromyxosis in turbot and gilthead sea bream [10, 17, 38].

The overexpression of *MASP3b* in the three organs suggests the involvement of lectin complement pathway in the immune response and of C-type lectins as pattern recognition receptors (PRRs) for *E. scophthalmi*. The latter would be also confirmed by the regulation of three genes encoding this type of lectins: *MRC1*, up-regulated in spleen and pyloric caeca; *CLEC4M* up-regulated in pyloric caeca and *CD209*, up-regulated in pyloric caeca and down-regulated in spleen. These results support the hypothesis on *E. scopthalmi* recognition by turbot advanced in previous studies, which investigated the presence of carbohydrate terminals in the parasite [13] and the effect of lectins on the attachment and invasion of intestinal epithelium [14].

C-type lectins receptors located on antigen-presenting cells play a major role in pathogen recognition and induction of immune response [39, 40], and *CD209*, in particular, is considered a marker for dendritic cells [41], key linkers between innate and adaptive immunity. Additionally, our study revealed the same expression pattern (up-regulation in pyloric caeca and down-regulation in spleen) of another dendritic cell marker, *CD83*. The presence and function of these cells in teleosts is largely unknown [42] and our findings provide additional evidence of their involvement in disease response in turbot [43]. The down-regulation of these genes in a main antigen-processing organ, like the spleen in teleost [44], together with previous observations [10, 11, 45] and other findings of this work, suggests that there might be a failure in the connection between innate and adaptive immune response during turbot enteromyxosis.

On this regard, it was remarkable to observe the down-regulation of several IFN-related genes in the three organs. Several type I IFN-induced genes and type II IFN receptors were depressed, and *PRDM1a*, a repressor of type II IFN [46], showed a five-fold increase in pyloric caeca. Interferons play a major role in signalling between innate and adaptive immune response [47, 48], and there are multiple lines of evidence that suggest their involvement in anti-parasitic response and resistance [17, 49, 50]. Notably, head kidney and spleen showed down-regulation of *TLR9*, a PRR which induces the expression of type I interferon via the action *IFR3* and *IFR7* (likewise down-regulated in these organs), and enhances MHC-I antigen (down-regulated in all organs) cross processing [50]. Although depression of MHC-I, IFNs and *TLR9* genes is commonly associated with immune system evasion by viruses [51–54], the importance of this mechanism in parasite infections is becoming increasingly evident [55–57]. In teleost, Young *et al.* [58] established the connection between the coordinated down-regulation of MHC-I and IFN-related genes in amoebic gill disease-affected Atlantic salmon and the inhibition of acquired immunity development and high susceptibility of this species to the disease. On the other hand, in gilthead sea bream chronically exposed to *E. leei*, IFNs and IFN-stimulated genes were hypothesized as markers for pathogen resistance due to their up-regulation in exposed but not parasitized fish [17].

Interestingly, our RNA-seq analysis also found a down-regulation of *IL*22 and *IL*17 that could reflect a decrease of their major producers, Th17 cells. Besides, *SOSCS*3, an inhibitor of *IL*17 expression, was up-regulated in spleen. Th17 cells also coordinate innate and adaptive immune response [59] and are described as critical for mucosal and epithelial host defence against extracellular pathogens [60]. The balance between the protective and harmful effects of Th17 cells through cytokines *IL17* and *IL22* is extremely delicate and there are reports supporting their contribution to host defence and others highlighting their inflammatory damage when the infection persists over time [60–63]. A deeper understanding of the genes underlying the activation or depression of the different T helper subsets responses is considered of great significance for resistance and tolerance of livestock species [64].

Nowadays, it is becoming evident that in a specific adaptive response is essential to deal with infection and to acquire resistance in teleost [45, 58, 65]. However these data points towards a possible failure or slowness in the activation of the adaptive response, as previously suggested for turbot enteromyxosis based on the evidence of delayed or even undetectable production of specific antibodies against *E. scophthalmi* by turbot [10, 45].

In this work, we found several down-regulated genes related to B and T cell activity in lymphohaematopoietic organs, also in accordance with previous work describing lymphocyte depletion, reduced Ig + cells and marked lymphohaematopoietic depletion in spleen and head kidney of turbot in advanced stages of the disease [8, 10, 11]. Globally, spleen and head kidney showed a depression of genes related to acquired immune response, which may reflect the incapability of the immune system to stop the infection. On the other hand, several immunoglobulin-related genes were up-regulated in pyloric caeca, which is consistent with the results of Bermúdez *et al.* [11], who found a progressive increase in IgM^+^ cells in the intestine of *E. scophthalmi*-infected turbot, reaching the maximum at 78 days post-exposure, suggesting that it may reflect a local reaction against the parasite. The importance of the local immune response has been recently highlighted for *E. leei*-infected gilthead sea bream [18], where significant changes in the expression of interleukins (ILs) and IL receptors were found in the intestine but not in head kidney nor in spleen of infected fish. In this organ, a switch from an early pro-inflammatory IL expression profile to an anti-inflammatory pattern in later stages of disease was also reported.

In our study, a remarkable number of up-regulated genes involved in immune and defence response were found in pyloric caeca, including several innate immune components involved in promoting the inflammatory reaction, which does not seem to be in a resolution phase. Also, intestinal-type alkaline phosphatase, an essential enzyme in controlling gut microflora and maintaining epithelial integrity, showed a -11.7 FC in infected turbot. The role of this enzyme in preventing intestinal inflammation has been demonstrated in zebrafish [66] and there is evidence of the beneficial effect of its administration in conditions of severe intestinal epithelial damage [67, 68]. This supports the hypothesis of an exacerbated local immune response of turbot against the parasite and its products [12, 15], which can explain the development of the observed lesions. The prolonged inflammation of pyloric caeca is likely to create an oxidative environment at the intestinal level, and the action of the inflammatory mediators and the oxidative stress can be responsible for the desquamation of the intestinal epithelial lining, typical of the disease in turbot, as seen in other conditions characterized by an exacerbated immune response [69–72]. In this context, beyong the up-regulation of two genes related to oxidative stress response (calcipressin 1 and adrenomedullin 5), the antioxidant defences appear to be failing, as revealed by the depression of the antioxidants SOD, CAT, MASRA, GSTT1 and TXNDC17. In chronic *E. leei*-infected gilthead sea bream, Davey *et al.* [17] found several genes encoding antioxidant enzymes up-regulated in response to the high reactive oxygen species production, so the opposite pattern reported here could also be one of the factors contributing to the higher susceptibility of turbot to enteromyxosis.

### Apoptosis and cell proliferation

Apoptosis is an essential biological process induced in response to many extrinsic stimuli (like inflammatory reaction and oxidative stress) and it can be considered as part of the host innate immune response during infection but also, in some cases, as an infection-associated immunopathology [73–76]. The ability of pathogen microorganisms, including intestinal parasites, to modulate apoptosis in their hosts has been widely documented [73, 77, 78]. We observed several DE genes related to apoptotic cell death in pyloric caeca of infected fish, including a considerable up-regulation of caspase-3. This is in accordance with previous histological observations describing increased number of cells with apoptotic features [8] and with the increase of active caspase-3 occurring in both lining epithelium and lamina propria of turbot intestine during enteromyxosis [16]. It has been suggested that this could be a mechanism used by the parasite to spread [8], though the observations of apoptotic cells between the inflammatory infiltrates of lamina propria have also been related to a strategy for immune evasion [16]. Our data also suggests that this process plays a prominent role in the pathogenesis of turbot enteromyxosis.

The up-regulation of some anti-apoptotic or apoptosis-induced genes (apoptosis inhibitor 5, *TNFRSF11B* and *HIGD1A*) and depression of some pro-apoptotic genes (*TRAIL* and *DEDD2*) was also detected at intestinal level, suggesting that complex adjustments in apoptotic signals may occur during enteromyxosis, as reported for *E. leei* [17]. These findings may be related to different requirements of the parasite to induce or inhibit apoptosis depending on the developmental stage, as hypothesized for human cryptosporidiosis [78]. Another possible explanation is that these genes may be counterbalancing the effects of the exacerbated immune response, also consistent with the activation of some tissue repair (up-regulation of *MMP13*, *PLAT* and *FGF10*) and cell proliferation (*PCNA* and cyclins) related genes observed in this organ.

Apoptotic cell death has also been involved in the lymphohaematopoietic depletion observed in infected turbot, either directly on cell components of these organs or indirectly as a result of the increment of leukocyte apoptosis in the intestine [8, 16]. In the current study, very few genes related to apoptosis are differently expressed in head kidney and spleen, displaying a substantial balance between cell death/survival signals. Concerning cell proliferation, the up-regulation in both organs of *HGF*, a pleiotropic cytokine that plays a major role in tissue regeneration, but also with potent anti-inflammatory properties, is a relevant observation, as it is involved in interfering the function of dendritic cells and CD4^+^ and CD8^+^ T cells [79–81]. Our results showed that CD4 and the dendritic cell markers (CD83, CD209) are down-regulated in spleen. Furthermore, *HGF* affects CD8^+^ cytotoxic cells by down-regulating IFN-gamma, granzyme and perforin [81], consistent with our results in head-kidney. The role of *HGF* in teleost is largely unknown, and in this case its activation may be an attempt to counterbalance the loss of cell population or to modulate the deleterious effects of an exacerbated immune response. These considerations need further research, in particular by focusing on the events occurring at earlier stages of the disease regarding cell-mediated immune response and the activation of interferon pathways at the site of infection as well as in lymphoid organs. In addition, more information about the changes in cell death/proliferation balance along the course of the disease will help to clarify the role of apoptosis in lymphoid depletion, and the conflict between host and parasite induced functions.

### Iron metabolism and erythropoiesis

Oxygen binding seems to be altered in both spleen and head kidney as shown by the gene-enrichment analysis performed, which strongly support alterations in the erythrocyte function and haemoglobin production. Both spleen and head kidney showed up-regulation of hepcidin, a peptidic hormone initially known by its antimicrobial activity, but later recognized as the principal regulator of iron homeostasis [82–84]. It acts as acute-phase protein to induce iron sequestration during infections and is considered the main gene responsible for the so called “anaemia of chronic disease” or “anaemia of infection” [82, 85]. Hepcidin determines a decreased absorption of iron in the intestine and sequestration of iron in macrophages, so limiting its availability for haemoglobin synthesis in maturing erythrocytes [82, 86]. RNA-seq analysis showed that in the lymphohaematopoietic organs several genes related to haemoglobin and erythrocytes maturation are markedly down-regulated as well as ferritin, the main iron-storage protein. This group of is genes is tightly clustered in the heatmap (Figure 3), revealing a strong common regulation. The depression of this group of genes can explain the decrease in hematocrit and haemoglobin values seen in *Enteromyxum*-infected fish [6] and the activation of genes related to the response to hypoxia found in this study (*HIGD1A*, cytochrome c oxidase and angiopoietin-related). The reduction in iron availability, however, may be explained both by the infection-related iron sequestration and the probable restricted iron intake by diet, due to the anorexia and the impaired intestinal absorptive function shown by fish suffering enteromyxosis [6, 87].

### Metabolism and digestive function

*Enteromyxum*-infected turbot have significantly lower weight and poorer condition at advanced stages of the disease [10], which is likely due to the reduction of food intake due to anorexia and intestinal damage [7, 8]. Effects of starvation have been shown to affect blood as well as immune function, besides the expected alterations in nutrient metabolism [88–90], as observed for the enriched GO category “lipid metabolism” in pyloric caeca in our study. Actually, in infected turbot we have found a remarkable decrease in the expression of several digestive enzymes in this organ, probably related to a general loss of the intestinal function as recently reported in cunner (*Tautogolabrus adspersus,* Walbaum) subjected to acute or long-term fasting [91].

Moreover, we observed changes in the expression of genes that encode for peptide hormones, which act in feeding behaviour. Gastrin cholecystokinin-like peptide and its related receptor *CCK2*, *GIP* and galanin receptor 1, were all down-regulated. Gastrin and cholecystokinin are structurally and functionally related hormones that act in response to food intake to stimulate different digestion processes. Similarly, gastric inhibitory polypeptide belongs to the incretin family of gastrointestinal hormones whose main function is to induce insulin secretion in response to the increase in the glucose blood level after food ingestion. The depression of these genes can be explained by the overall reduction of digestive function caused by food deprivation, as postulated by Hayes & Volkoff [91] for cholecystokinin decreased expression seen in fasting cunner. On the other hand, galanin is an orexigenic hormone and its decrease can be related to the parallel increase of the anorexigenic *CART* and *CGRP*.

Anorexia is a quite common clinical sign observed in parasitic infection but the causes and significance that underlie this behaviour, despite being investigated for many years, are still controversial [92–94]. In *E. leei*-infected gilthead sea bream, Estensoro *et al.* [87] found evidence that anorexia is the main cause of body mass loss, hypothesizing the involvement of cachectic cytokines, gastrointestinal peptides and growth factors in the voluntary reduction of food intake. The results of our study are consistent with these considerations, although further investigation of the mechanisms underlying anorexia in enteromyxosis should be addressed.

### Cytoskeleton and extracellular matrix

RNA-Seq analysis revealed that the three studied organs shared common depression of numerous genes encoding cytoskeletal and structural proteins. Protein synthesis is an energy-demanding process and can be affected by the detrimental effects of a prolonged reduction in food intake, further exacerbated if it is associated with impaired intestinal absorption [95, 96]. These changes can therefore be considered as indicative of a progressive overall tissue wasting which leads to the lethal outcome of turbot enteromyxosis.

## Conclusions

This is the first application of RNA-seq technology to the study of turbot transcriptomic response and particularly to the analysis of *E. scophthalmi* infection. This experiment has greatly enriched our knowledge on the major turbot biological processes and responses against this disease. The results obtained point towards the presence of an exacerbated local immune response associated with an inadequate activation of the adaptive immunity, probably related to the failure of some components acting in bridging innate and adaptive immune response. Additionally, the involvement of C-type lectins as PRR for the parasite and of apoptosis in the pathogenic mechanism is highly plausible. The transcriptomic analysis has also revealed details on the genetic basis underlying the characteristic clinical signs and lesions associated with the progression of this disease, like cachexia and anaemia. This knowledge is essential to investigate the pathogenetic mechanisms and the differences in species-specific susceptibility to enteromyxosis with the aim of identifying resistance-related genes. This information will be useful for the derivation of new therapeutic treatments and to exploit genetic variation associated with these key genes in order to achieve more resistant broodstock through breeding programmes. Further analyses should focus on transcriptomic changes at earlier stages of the disease and on comparative studies with other affected species.

## Methods

### Experimental design and animal sampling

Turbot (150 g mean weight) were obtained from an *E. scophthalmi-*free farm in northwestern Spain and kept in the facilities of the Instituto de Acuicultura de Torre la Sal (IATS, Cabanes, Castellón, Spain). Animals were divided into 55 RCPT and 65 CTRL fish, and were acclimated for two weeks under identical conditions before starting the trial in two 500 L tanks per group with 5 μm-filtered and UV-irradiated open flow sea water (37.5‰ salinity) at 19 ± 1°C.

The experimental infection was carried out by oral route, as described by Redondo *et al.* [97]. Briefly, RCPT fish received 1 ml of intestinal scraping homogenates in Hank’s Balanced Salt Solution (HBSS) from 20 donor fish, containing *E. scophthalmi* live parasites, whereas CTRL fish were inoculated with the same amount of HBSS alone. Donor turbot came from an experimentally infected stock maintained at IATS.

Fifteen RCPT and 10 CTRL fish were sampled at 7, 24 and 42 days post-inoculation (DPI) in order to obtain a representative time-course that included different levels of infection for analysis. At each sampling point, fish were euthanized under benzocaine anaesthesia (3-aminobenzoic acid ethyl ester, 100 mg/ml) (Sigma, St. Louis, MO, USA) and necropsied to obtain samples from head kidney, spleen and digestive tract.

The experiment was carried out in accordance with national (Royal Decree RD1201/2005, for the protection of animals used in scientific experiments) and institutional regulations (CSIC, IATS Review Board). Animals were treated according to the Directive 2010/63/UE of the European Parliament and of the Council of 22 September 2010 on the protection of animals used for experimentation and other scientific purposes. All experimental protocols were approved by the Institutional Animal Care and Use Committee of the University of Santiago de Compostela (Spain).

### Histopathology

Tissue samples were fixed in Bouin’s fluid at 4°C for 12 hours and then stored in 70% ethanol until being processed for paraffin-embedding. Thin sections (3 μm) were stained with H&E and toluidine blue for microscopic evaluation. The healthy status of CTRL fish was confirmed while RCPT fish showed a variable level of infection irrespective of the day of sampling, suggesting different resistance to the parasite or variation associated to the infection protocol. Consequently, fish were classified according to the lesional degree in three groups (slight, moderate and severe), as described by Bermúdez *et al.* [8].

### RNA extraction and sample preparation for RNA-seq

Samples from the three organs (head kidney, spleen and pyloric caeca) were collected in cold RNAlater (Qiagen), kept at 4°C overnight and then transferred to -20°C. RNA extraction was performed using the RNeasy mini kit (Qiagen) with DNase treatment following manufacturer’s instructions. RNA quality and quantity were evaluated in a Bioanalyzer (Bonsai Technologies) and in a NanoDrop® ND-1000 spectrophotometer (NanoDrop® Technologies Inc), respectively. Prior to sequencing, RNA aliquots from three CTRL fish were pooled by organ, while samples from three RCPT fish were analyzed individually, resulting in three RCPT and one CTRL sample per organ. The three RCPT fish were chosen among those fish graded as severely infected in histopathology assessment and were collected at the same time, thus, both RCPT and CTRL fish belonged to the 42 DPI sampling point.

The 12 samples were barcoded and prepared for sequencing by the Wellcome Trust Centre for Human Genetics, Oxford, using standard protocols. Sequencing was conducted on an Illumina HiSeq 2000 as 100 bp paired-end reads.

The quality of the sequencing output was assessed using FastQC (http://www.bioinformatics.babraham.ac.uk/projects/fastqc/; version 0.10.1). Quality filtering and removal of residual adaptor sequences was conducted on read pairs using Trimmomatic [98] (version 0.30). Specifically, residual Illumina specific adaptors were clipped from the reads, leading and trailing bases with a Phred score less than 4 were removed and the read trimmed if a sliding window average Phred score over four bases was less than 15. Only reads where both pairs had a length greater than 36 bp post-filtering were retained, leaving on average more than 15 million mappable paired-end reads per sample.

The recently assembled turbot genome (Figueras *et al.*, unpublished data), was used as a reference for read mapping. The genome consists of 16,493 scaffolds with an N50 of 4,268,014 bp and N90 of 462,971 bp. Filtered reads were mapped to the genome using Tophat2 [99] (version 2.09) that leverages the short read aligner Bowtie2 [100] (version 2.1.0) with a maximum intron length of 10 kb. HTSeq-count (http://www-huber.embl.de/users/anders/HTSeq/doc/overview.html) was used to extract the raw reads from the mapping files.

To account for differences in the mass composition of the RNA-Seq samples, we conducted trimmed mean of M-values (tmm) normalisation of expression values using EdgeR [101] for each organ. Differential expression between infected and control samples was calculated using EdgeR and resulting *P-*values corrected for false discovery rate (FDR). DE genes were defined as showing an FDR corrected *P*-value < 0.05, a log_2_ fold FC >1 and a minimum length of 200 bp. The DE genes were identified and annotated using Blast2GO (version 2.7.0) with an E-value cutoff of E^-6^. Enriched GO terms for each tissue were identified by comparing the DE genes against the full turbot transcriptome using Blast2GO Fisher’s exact test (p < 0.05, FDR corrected). The correct classification of the samples as either infected treatments (clustering together) or uninfected controls (as outgroups) was confirmed with hierarchical clustering implemented in the R package “pvclust” [102], using complete Euclidean distance with 1000 bootstrap replicates.

The data discussed in this publication have been deposited in NCBI’s Gene Expression Omnibus [103] and are accessible through GEO Series accession number GSE63911 (http://www.ncbi.nlm.nih.gov/geo/query/acc.cgi?acc=GSE63911).

### List of gene symbols

*IL4I1*: L-amino-acid-oxidase; *ALOXE3*: epidermis-type lipoxygenase 3; *MASP3b*: mannose-binding lectin-associated serine protease-3b; *IRF 3, 4, 7*: interferon regulatory factor; *Gig2*: grass carp reovirus-induced gene 2; *IFIT-1*: interferon-induced protein with tetratricopeptide repeats 1; *cHERC5*: e3 isg15--protein ligase herc5; *IFI44*: interferon-induced protein 4; *CAT*: catalase; *FMNL1*: formin-like protein 1;*TMOD4*: tropomodulin 4; *GRXCR1*: Glutaredoxin domain-containing cysteine-rich protein 1; *SNPH*: syntaphilin-like isoform ×1; *COL1A2*: collagen alpha-2 chain; *KCNN3*: small conductance calcium-activated potassium channel protein 3-like; *EGR*: early growth response; *TNFRSF10b*: tumor necrosis factor receptor superfamily, member 10b; *G-CSFR*: granulocyte colony-stimulating factor receptor; *AP-1*: transcription factor ap-1; *CEBPB*: ccaat enhancer-binding protein beta; *LITAF*: lipopolysaccharide-induced tumor necrosis factor-alpha factor; *CYBB*: cytochrome b-245 heavy chain; *TNF-α*: tumor necrosis factor-alpha; *TCRB*: t-cell receptor beta chain; *MRC1:* macrophage mannose receptor 1*; MASP1:* mannan-binding lectin serine protease 1*; CLEC4M:* C-type lectin domain family 4, member M*; Gig1*: grass carp reovirus-induced gene 1; *IFNGR1*: interferon gamma receptor alpha chain; *GVINP1*: interferon-induced very large gtpase 1; *TLR9*: toll-like receptor 9; *IL 22, 17*: interleukin; *MRSA*: peptide methionine sulfoxide reductase; *SOD*: superoxide dismutase*; GSTT1*: glutathione s-transferase theta-1; *TXNDC17*: thioredoxin domain-containing protein 17; *MMP13*: collagenase 3; *PLAT*: tissue-type plasminogen activator; *FGF10*: fibroblast growth factor 10, *VNT*: vitronectin; *HGF*: hepatocyte growth factor; *HGFR*: hepatocyte growth factor receptor; *HGFAC*: hepatocyte growth factor activator; *HIGD1A*: hig1 domain family member 1a; *PCNA*: proliferating cell nuclear antigen; *TAL1*: t-cell acute lymphocytic leukemia protein 1; *GATA2*: gata-binding factor 2; *TPM4*: tropomyosin alpha-4 chain; *GIP*: gastric inhibitory polypeptide; *CCK2*: cholecystokinin B receptor; *CART*: cocaine- and amphetamine-regulated transcript; *CGRP*: calcitonin gene-related peptide; *PRDM1a*: *PR* domain containing 1a, with ZNF domain; *SOSCS*3: suppressor of cytokine signalling 3; *TNFRSF11B*: tumor necrosis factor receptor superfamily, member 11b; *TRAIL*: TNF-related apoptosis-inducing ligand; *DEDD2*: DNA-binding death effector domain-containing protein 2.

## Electronic supplementary material

Additional file 1: Table S1: List of shared differentially expressed genes between the three organs. Common differentially expressed genes between kidney, spleen and pyloric caeca. Fold change by tissue, fragments per kilobase of exon per million fragments mapped (FPKM) in control and infected samples by tissue and GO terms for each gene are shown. (XLSX 22 KB)

Additional file 2: Table S2: List of differentially expressed genes in head kidney. Differentially expressed genes in kidney. Fold change, fragments per kilobase of exon per million fragments mapped (FPKM) in control and infected samples and associated functional group for each gene are shown. (XLSX 16 KB)

Additional file 3: Table S3: List of differentially expressed genes in spleen. Differentially expressed genes in spleen. Fold change, fragments per kilobase of exon per million fragments mapped (FPKM) in control and infected samples and associated functional group for each gene are shown. (XLSX 16 KB)

Additional file 4: Table S4: List of differentially expressed genes in pyloric caeca. Differentially expressed genes in pyloric caeca. Fold change, fragments per kilobase of exon per million fragments mapped (FPKM) in control and infected samples and associated functional group for each gene are shown. (XLSX 21 KB)
